# Systematic review protocol for complications following surgical decompression of degenerative cervical myelopathy

**DOI:** 10.1371/journal.pone.0296809

**Published:** 2024-01-29

**Authors:** Esmaeil Mohammadi, Ali Fahim Khan, Lance M. Villeneuve, Sanaa Hameed, Grace Haynes, Fauziyya Muhammad, Zachary A. Smith

**Affiliations:** 1 Department of Neurosurgery, University of Oklahoma Health Sciences Center, Oklahoma City, Oklahoma, United States of America; 2 Neuroscience Program, University of Oklahoma Health Sciences Center, Oklahoma City, Oklahoma, United States of America; 3 Stephenson School of Biomedical Engineering, University of Oklahoma, Norman, Oklahoma, United States of America; Duke University Medical Center: Duke University Hospital, UNITED STATES

## Abstract

**Background:**

Degenerative Cervical Myelopathy (DCM) is one of the most common degenerative disorders of cervical spine and sources of cord dysfunction in adults. It usually manifests with neurologic presentations such as loss of dexterity and gait issues. Treatment for moderate and severe cases of DCM is surgical decompression of the region. There are many approaches available for surgical intervention which could be categorized into anterior and posterior based on the side of neck where operation takes place. Additionally, for certain cases the hybridized anterior-posterior combined surgery is indicated. While there are many technical differences between these approaches with each having its own advantages, the complications and safety profiles of them are not fully disclosed. This protocol aims to systematically search for current reports on complications of surgical decompression methods of DCM and pool them for robust evidence generation.

**Method:**

Search will be carried out in PubMed, Scopus, and Cochrane databases for retrospective and prospective surgical series, cohorts, or trials being performed for DCM with at least a sample size of 20 patients. Query strings will be designed to capture reports with details of complications with no year limit. Studies not being original (e.g., review articles, case reports, etc.), not in English, having patients younger than 18-years-old, and not reporting at least one complication will be excluded. Two independent reviewers will review the titles and abstracts for first round of screening. Full text of retrieved studies from previous round will be screened again by the same reviewers. In case of discrepancy, the third senior reviewer will be consulted. Eligible studies will then be examined for data extraction where data will be recorded into standardized form. Cumulative incidence and 95% confidence intervals of complication will be then pooled based on generalized linear mixed models with consideration of approach of surgery as subgroups. Heterogeneity tests will be performed for assessment of risk of bias.

**Discussion:**

This systematic review is aimed at providing practical information for spine surgeons on the rates of complications of different surgical approaches of DCM decompression. Proper decision-making regarding the surgical approach in addition to informing patients could be facilitated through results of this investigation.

## Introduction

Cord compression following degenerative processes in cervical spine often leads to Degenerative Cervical Myelopathy (DCM) also known as cervical spondylotic myelopathy, a common cause of myelopathy in adults. It is associated with cervical spinal cord tissue injury [1] and functional changes in the brain [2]. DCM commonly presents with neurologic manifestations such as loss of dexterity and gait issues [3] and patients are typically screen using Magnetic Resonance Imaging [4]. Non-operative management for mild cases and surgical decompression for moderate and severe cases at the stenotic areas are typically chosen. However, the natural history of DCM demonstrates neurological deterioration over the subsequent course of disease, indicating surgery for most cases [5, 6]. Debate currently remains for the ideal decompressive intervention with options including anterior, posterior, and a combined anterior-posterior approaches.

The existing evidence on the incidence of complications in different approaches to DCM decompression is inconclusive and remarkably varied [7]. These varying profiles are significant because complications such as esophageal perforation are much more severe, requiring extended hospitalization and extensive medical care. This protocol tries to describe the perioperative complications of DCM surgeries and to assist surgeons in decision-making and patient counseling.

## Materials and methods

We will perform an aim-directed systematic review of cross-sectional, cohort, and trial studies to achieve the rate of complications of DCM decompression surgeries. We will follow Preferred Reporting Items for Systematic Reviews and Meta-Analysis (PRISMA) [8] for reporting results and flow of searched studies. Protocol version of this checklist (PRISMA-P [9]) is provided in S1 Table.

### Eligibility criteria

To be eligible, studies should be peer-reviewed works in English with either retrospective or prospective cross-sectional, cohort, or trial designs and enroll at least 20 conventional surgical cases older than 18 years with reports of at least one complication (Table 1). Conventional surgery approaches were chosen as: 1- anterior decompression with/without fixation, 2- posterior decompression with/without fixation, 3- anterior decompression with fixation combined with posterior fixation. In order to make the synthesis and analysis more applicable to everyday medical practice, we used a previous review of cervical spine surgeries [10] and focused on the most commonly encountered major complications (Table 2).

**Table 1:** Eligibility based on population, intervention, control and outcomes (PICO) structure.

Population	DCM patients older than 18-years
Intervention	Anterior, posterior, or hybrid anterior-posterior surgeries
Comparator	DCM patients without complications of interest
Outcome	Perioperative complications
Study design	Retrospective or prospective cross-sectional, cohort, or trial studies with at least 20 surgical cases with no year limit

**Table 2:** Definition of complications.

Complication	Definition
Dysphagia	Injury to the pharynx or esophagus, or postoperative difficulty swallowing
Cerebrospinal fluid (CSF) leak	Leakage of fluid and/or documented tear in the protective membrane covering the spinal cord
Wound complications	Surgical site infection or wound opening
Neurological damage	Injury to the spinal cord or nerve roots, or postoperative weakness in the upper limbs
Dysphonia	Injury to the voice box, laryngeal nerves, or postoperative hoarseness
Hemorrhagic events	Injury to blood vessels during surgery or observation of a growing hematoma deep within the wound
Coagulation events	Deep venous thrombosis, pulmonary embolism, or non-posterior ischemic strokes
Pneumonia	Infections of the respiratory tract
Urinary tract infection (UTI)	Infections of the urinary system
Reoperation	Indication of reoperating on same patient for reasons such as hardware failure, revision surgery, or other complications management
Readmission	Readmission to hospital after being discharged

### Information sources

PubMed, Scopus, and Cochrane will be systematically searched to identify any eligible peer-reviewed work. Cross-reference checks will be performed for included studies with manual investigation of Google Scholar. Rayyan (ryyan.ai) platform will be utilized for screening procedure and duplicates removal.

### Search strategy

Experienced researchers in systematic reviews will design search strategies incorporating keywords DCM and its related derivatives, with consideration of different DCM surgeries and associated complications. These strategies will be tailored to different databases to maximize the effectiveness of the search. To the search to include only human studies, filters will be utilized to exclude any research involving non-human animals. PubMed’s search strategy is shown in S1 File.

### Study selection

Two independent reviewers will go over the titles and abstracts of retrieved works and perform the first round of screening in Rayyan tool. In the next step, a second round of screening will be performed based on the full text of potentially eligible studies against the eligibility criteria. In case of discrepancies, a third senior reviewer will be consulted. PRISMA flowchart will be illustrated to depict the flow of documents and reasons for exclusion.

### Data extraction

The same two independent reviewers will do the extraction process into a predefined standard form (S2 Table). The extraction form will be created based on piloted studies. In a situation where disagreement arises the third senior reviewer will be questioned. The authors of original works will be reached out if any question or ambiguity of data is encountered. The extraction form is represented in the appendix.

### Data items

Data such as the first author, year of publication, design, location of study, sample size with sex ratio, age, approach of surgery, number of spinal levels, complication and designated rate will be extracted. For studies using databases, the name of database will also be extracted.

### Risk of bias

As we anticipate including majority of works from non-randomized works, the Joanna Briggs Institute’s (JBI) tool for assessment of risk of bias in cohort and cross-sectional studies will be used. Same independent reviewers will carry out the risk assessment and the third senior reviewer will be consulted wherever deemed necessary.

### Outcomes

Perioperative complications of DCM decompression will serve as outcomes of interest. The full list of complications with their definitions is provided in Table 2.

### Summary measures

The pooled incidence rates of each complication with estimated 95% confidence interval will be reported. Moreover, these values will be generated for anterior, posterior, and combined anterior-posterior approaches for purpose of comparison.

### Synthesis of results

Incidence of complications will be aggregated into cumulative rate with 95% confidence interval. We calculated Event counts will be rounded to the closest integer The generalized linear mixed model (GLMM) with logit transformation (also known as *‘PLOGIT’*) of proportions will be used for the purpose of pooling. The Clopper-Pearson’s method will be utilized for confidence interval estimation. Random-effect approach will be used for all analyses. We will not perform imputation of missing values while missing items will be disregarded from specific analyses. All analyses will be performed using R statistical packages for meta-analysis. I^2^ statistics will be used for heterogeneity assessment with values more than 50% as heterogenous. The subgroup analysis will be used to compare approaches of surgery. Between-group comparison will be done by one-way ANOVA or Chi-square test, whichever appropriate. Two-sided alpha levels less than 0.05 will be regarded as significant.

### Risk of publication bias

This form of bias will be checked by investigation of funnel plots via Egger’s test of funnel asymmetry.

### Ethics

As secondary published data are going to be used, ethics approval is not required.

## Discussion

### Expected benefits

Many DCM patients undergo cervical decompression surgeries nowadays. This systematic review and meta-analysis will shed light on the rate of complications of DCM surgeries and compare main surgical methods allowing patients and spine surgeons to make more informed decisions.

### Limitations

Anterior and posterior surgical approaches commonly are indicated for different pathology variants that can differ substantially and combining them could make interpretation difficult. Moreover, we anticipate a high degree of heterogeneity in the subsequent samples. We will perform subgroup analysis of different approaches to overcome these limitations to some extent.

### Conclusion

This review will be a comprehensive assessment of current state of literature on cons of spine surgeries and build a foundation for informed clinical decision making and also to identify potential areas of improvement in both research and clinical arenas.

## Supporting information

S1 Table PRISMA-P checklist. (PDF)

S2 Table Data extraction form. (XLSX)

S1 File Search strategy for PubMed. (DOCX)

## References

[1] Khan AF, Mohammadi E, Haynes G, Hameed S, Rohan M, Anderson DB, et al. Evaluating tissue injury in cervical spondylotic myelopathy with spinal cord MRI: a systematic review. European Spine Journal. 2023 Nov 5:1–22. doi: 10.1007/s00586-023-07990-0 37926719 PMC13274496

[2] Khan AF, Muhammad F, Mohammadi E, O’Neal C, Haynes G, Hameed S, et al. Beyond the aging spine–a systematic review of functional changes in the human brain in cervical spondylotic myelopathy. GeroScience. 2023 Oct 6:1–30. doi: 10.1007/s11357-023-00954-8 37801201 PMC10828266

[3] Brain WR, Northfield D, Wilkinson M. The neurological manifestations of cervical spondylosis. Brain. 1952;75: 187–225. doi: 10.1093/brain/75.2.187 14934989

[4] Khan AF, Haynes G, Mohammadi E, Muhammad F, Hameed S, Smith ZA. Utility of MRI in Quantifying Tissue Injury in Cervical Spondylotic Myelopathy. Journal of Clinical Medicine. 2023 May 8;12(9):3337. doi: 10.3390/jcm12093337 37176777 PMC10179707

[5] Karadimas SK, Erwin WM, Ely CG, Dettori JR, Fehlings MG. Pathophysiology and natural history of cervical spondylotic myelopathy. Spine. 2013;38: S21–S36. doi: 10.1097/BRS.0b013e3182a7f2c3 23963004

[6] Martin AR, Kalsi-Ryan S, Akbar MA, Rienmueller AC, Badhiwala JH, Wilson JR, et al. Clinical outcomes of nonoperatively managed degenerative cervical myelopathy: an ambispective longitudinal cohort study in 117 patients. J Neurosurg Spine. 2021;34: 821–829. doi: 10.3171/2020.9.SPINE201395 33836502

[7] Mummaneni PV, Kaiser MG, Matz PG, Anderson PA, Groff MW, Heary RF, et al. Cervical surgical techniques for the treatment of cervical spondylotic myelopathy. J Neurosurg Spine. 2009;11: 130–141. doi: 10.3171/2009.3.SPINE08728 19769492

[8] Page MJ, McKenzie JE, Bossuyt PM, Boutron I, Hoffmann TC, Mulrow CD, et al. The PRISMA 2020 statement: an updated guideline for reporting systematic reviews. Syst Rev. 2021;10: 89. doi: 10.1186/s13643-021-01626-4 33781348 PMC8008539

[9] Moher D, Shamseer L, Clarke M, Ghersi D, Liberati A, Petticrew M, et al. Preferred reporting items for systematic review and meta-analysis protocols (PRISMA-P) 2015 statement. Syst Rev. 2015;4: 1. doi: 10.1186/2046-4053-4-1 25554246 PMC4320440

[10] Cheung JPY, Luk KD-K. Complications of Anterior and Posterior Cervical Spine Surgery. Asian Spine J. 2016;10: 385–400. doi: 10.4184/asj.2016.10.2.385 27114784 PMC4843080

