# Supplementary material for: Systematic review protocol for complications following surgical decompression of degenerative cervical myelopathy
Source: PLoS One. 2024 Jan 29;19(1):e0296809. doi: 10.1371/journal.pone.0296809 (PMC10824432; doi:10.1371/journal.pone.0296809)
Supplement: S1 File — (DOCX) [file pone.0296809.s003.docx]

PubMed

"complicat*"[All Fields] OR "readmi*"[All Fields] OR "mortal*"[All Fields] OR "morbid*"[All Fields]

OR "fail*"[All Fields] OR "revis*"[All Fields] OR "pseudoarthro*"[All Fields]) AND (("cervical

spondylotic myelopathy"[Title/Abstract] OR ("degenerative spondylotic myelopathy"[Title/Abstract]
